# A Qualitative Exploration of Social Contact Patterns Relevant to Airborne Infectious Diseases in Northwest Bangladesh

**DOI:** 10.3329/jhpn.v31i4.19976

**Published:** 2013-12

**Authors:** Sabiena G. Feenstra, Quamrun Nahar, David Pahan, Linda Oskam, Jan Hendrik Richardus

**Affiliations:** ^1^Erasmus MC, University Medical Center Rotterdam, Department of Public Health, Rotterdam, The Netherlands; ^2^Centre for Population, Urbanization and Climate Change, icddr,b, GPO Box 128, Dhaka 1000, Bangladesh; ^3^Rural Health Program Nilphamari, The Leprosy Mission International Bangladesh, Dhaka, Bangladesh; ^4^KIT Biomedical Research, Amsterdam, The Netherlands

**Keywords:** Airborne infectious diseases, Leprosy, Social contact patterns, Tuberculosis, Bangladesh

## Abstract

In South Asia, the burden of infectious diseases is high. Socioeconomically and culturally-defined social interaction patterns are considered to be an important determinant in the spread of diseases that are transmitted through person-to-person contact. Understanding of the contact patterns in this region can be helpful to develop more effective control measures. Focus group discussions were used in exploring social contact patterns in northwest Bangladesh. The patterns were assessed for perceived relevance to the spread of airborne infectious diseases, with special focus on diseases, like leprosy and tuberculosis, in which the role of social determinants is well-recognized. Highly-relevant social contact patterns inside the home and the neighbourhood, across age and sex groups, were reported in all group discussions. Outside the home, women and girls reported relevant contacts limited to the close neighbourhood while men mentioned high relevant contacts beyond. This implies that, in theory, infectious diseases can easily be transmitted across age and sex groups in and around the home. Adult men might play a role in the transmission of airborne infectious diseases from outside this confined area since only this group reported highly-relevant social contacts beyond the home. This concept needs further exploration but control programmes in the South Asian region could benefit from considering differences in social contact patterns by gender for risk assessments and planning of preventive interventions.

## INTRODUCTION

The South Asian region has a high burden of infectious diseases. These diseases form a leading cause of morbidity and mortality and are responsible for 42% of all disability-adjusted life years (DALYs) in the region (1,2). Socioeconomic, environmental and behavioural factors contribute to the high prevalence of infectious diseases. The situation in the region is worsened by considerable poverty, prevailing inequities, poor health infrastructure, and inability to allocate resources for public health (3,4).

Some infectious diseases seem to be influenced more by the social environment than others. Leprosy and tuberculosis are examples of diseases in which the role of social determinants is well-recognized (5,6). Leprosy and tuberculosis are still endemic in the poorest countries of the world and, within these countries, especially in the poorest regions or urban slums (7,8). Although a causal relationship between poverty and these diseases is difficult to demonstrate, socioeconomic determinants have been suggested to be of major influence on the continuing transmission of these infectious diseases (6,9,10).

Interactions among people through social contacts play an essential role in the spread of infectious diseases, like tuberculosis and leprosy transmitted from person to person (11-14). Both diseases are caused by *Mycobacteria* that use an airborne route by small droplets as main mode of transmission while intensive or longstanding contacts with a patient are required (8,15). Determinants, like social, economic and cultural factors, are of influence on the number and variety of social contacts, and on the intensity and duration of these contacts. These aspects are important in the spread of infectious diseases (16,17).

In most Asian countries, only limited information is available on how people interact and which social contact patterns are important for the spread of infectious diseases. The understanding of specific social contact patterns in this region can be helpful to develop more effective control measures for infectious diseases. Bangladesh is one of the poverty-stricken countries in this region where limited information is available on social contacts and the way people interact while the burden of infectious diseases, including tuberculosis and leprosy, is high. In a recent nationwide survey (2007/2009), the prevalence of tuberculosis was 79 per 100,000 inhabitants but much higher rates were detected among the poor and uneducated population groups (18). Although the registered prevalence of leprosy in Bangladesh has been below 1.0 per 10,000 inhabitants over the last decade, a survey in northwest Bangladesh revealed 15.1 cases of previously-undiagnosed leprosy per 10,000 inhabitants in 2002/2003 (19).

The objective of this study was to explore social contact patterns in northwest Bangladesh in order to identify behavioural patterns that could facilitate spread of infectious diseases. We placed a special focus on contact patterns that are important for the spread of airborne diseases, such as tuberculosis and leprosy, in which the social environment plays an important role.

## MATERIALS AND METHODS

### Study design

We used a qualitative method with focus group discussions to explore social contact patterns in northwest Bangladesh. This allowed us to elaborate on social contact patterns without predefined assumptions and to identify a broad range of ideas on the topic in a relatively short time.

### Study area

The study was carried out in Nilphamari and Rangpur districts in northwest Bangladesh. In this predominantly rural area, which is one of the poorest parts of the country, leprosy and tuberculosis are endemic (20,21). We conducted focus group discussions in two villages and one urban ward. The locations were selected in collaboration with field staff of the Rural Health Program (RHP) of The Leprosy Mission International Bangladesh (TLMB), familiar with the region. We selected three areas with different living circumstances to be able to explore a variety of social contacts and local habits: an urban area in one of the main cities, a village proximate to a town, and a village without good access to the nearest town.

### Study population

In each of the three study areas, we formed four discussion groups, each with 10-12 healthy individuals, homogeneous for sex and age (Table 1). An RHP volunteer supported the logistic management of the focus group discussions and recruited the participants. Since it was not possible to make homogeneous groups in terms of religion and socioeconomic background, we ascertained that, in all groups, the two main religions in the region (Muslim and Hindu), and different socioeconomic backgrounds were represented since these factors were expected to have influence on social contact patterns. In the two rural locations, male participants were mostly engaged in farming whereas, in the urban area, they had a variety of jobs ranging from day-labour to small business. Most female participants were housewives. In rural areas, however, all females were also involved in farming.

### Data collection

The focus group discussions took place in March 2009. Staff members of RHP, experienced in conducting group meetings, facilitated the focus group discussions, together with a social scientist. Before the start of the study, the RHP staff received training and written guidelines in Bangla and English.

We organized the focus group discussions at an easy-to-reach location within the selected villages and urban ward. Due to cultural reasons, the discussions with women took place in a yard with a fence or wall while, for the discussions with men, an easy-to-reach outside location was chosen. An RHP staff member and a local volunteer assured the privacy by guarding the area and sending bystanders away.

Participants and staff members were seated at the same level to create a good atmosphere for discussion and sharing of ideas and to minimize the hierarchy between researchers and participants. For good rapport, the female discussion groups were facilitated by a female staff member and male discussions by a male one. All meetings lasted for about one hour. The discussions were held in the local language (Bangla) and recorded with tape-recorders.

**Table 1.** Characteristics of participants in the focus group discussions

Site	Group	N	Age (years)	Religion		Years of education		
			Average (min-max)	Muslim (%)	Hindu (%)	0 (%)	1-5 (%)	5+ (%)
Urban	Adult male	11	44 (30-70)	73	27	18	36	46
	Adult female	11	38 (25-60)	64	36	0	45	55
	Adolescent boys	11	19 (17-20)	45	55	18	55	27
	Adolescent girls	12	18 (15-20)	50	50	0	0	100
Rural 1	Adult male	12	44 (30-70)	58	42	33	17	50
	Adult female	12	35 (25-45)	50	50	58	17	25
	Adolescent boys	12	16 (11-20)	50	50	0	33	67
	Adolescent girls	11	15 (12-17)	36	64	0	55	45
Rural 2	Adult male	12	39 (30-60)	42	58	42	25	33
	Adult female	11	42 (35-65)	25	75	100	0	0
	Adolescent boys	11	18 (16-20)	42	58	9	27	64
	Adolescent girls	10	14 (13-16)	0	100	0	20	80
		136		45	55	24	27	49

We developed a list of topics to structure the focus group discussions. Social contacts were studied at three different levels (home, neighbourhood, and outside the neighbourhood) taking the household as core functional unit in Bangladesh society (22,23), i.e. centre of the social contact structure and assuming that the intensity of social contact decreased with distance from the core unit. Social contact was defined as all contacts among people. This could, thus, range from a short conversation on the street to close physical contact when sleeping with someone in the same bed. This broad definition was used in exploring social contacts in this region without predefined assumptions.

Although the list of topics was used for structuring the discussion, we instructed the facilitators to start with open questions and to give participants the opportunity to raise new issues as well. Some examples of issues raised were seating arrangements in schools and attendance in religious festivals.

To pre-test the list of topics and obtain an expert opinion on the subject, we organized a group discussion for staff members from different programmes of TLMB in Nilphamari before the start of the study. In that meeting, five male and five female staff members with extensive field experience with leprosy patients participated. Several important issues were raised in that discussion, which needed in-depth exploration during the focus group discussions in the field. Examples are the potential role of hawkers and the importance of watching television in groups.

### Analysis

The staff members who facilitated the focus group discussions transcribed the recorded interviews in Bangla. Afterwards, the transcriptions were translated into English under the supervision of the social scientist. The software program N-vivo was used for conducting a thematic analysis (24).

We used two steps in the analysis of the data. The first step was to identify all different types of contacts with other people as reported in the focus group discussions and order them by themes. This is presented as a description supplemented by quotes of group members. The next step was to judge the relevance for transmission of airborne diseases of all contact patterns identified.

We developed a framework with three categories of perceived relevance for transmission: high, medium or low relevance, based on two important factors for the transmission of an airborne disease: intensity and duration of the contact (16,17,25). Based on this information, we assumed that contacts with other people in a small closed room were more intensive than contacts in an open (outside) area or a large well-ventilated room while contacts in which physical distance from one person to another was short (less than 1 metre) or in which direct skin-to-skin contact was involved were more intensive than contacts in which distance was kept. We assumed that contacts with the duration of more than 4 hours had the same relevance than contacts of long duration while regular short contacts (at least daily) were thought to be of high relevance for transmission as well.

We ordered all different contact patterns mentioned by the groups according to the framework. Patterns which were both intensive and of a long duration were classified as highly relevant for transmission. Contact patterns which scored positive on only one of these factors were classified as being medium relevant while patterns that were not intensive and of a short duration were classified as less relevant. Using the framework, we subsequently analyzed contact patterns mentioned by the different subgroups: men, women, adult, adolescent, rural, and urban.

### Ethical considerations

Before organizing the focus group discussions, we obtained consent from the village leaders. At the start of the discussion, we explained the subject, purpose, and procedures of the meeting to the participants, after which they gave their verbal consent for the discussion. A guardian was asked to give consent for those under the age of 16 years. Ethical approval for the study was obtained from the Bangladesh Medical Research Council under reference BMRC/NREC/2007-2010/2107.

## RESULTS

### Social contacts in the home

All groups reported that, in the home, household members, relatives, and neighbours from different ages and sex groups visited one another regularly. The participants reported that households in that area had an average size of five to seven people and consisted of parents, children, and sometimes grandparents or other relatives. They mentioned that most houses in rural areas consisted of one to five separate small houses made of bamboo and mud, functioning as separate rooms around a courtyard. Some houses were constructed with bricks or concrete. The latter types were more common in urban areas where houses had larger rooms but smaller or no courtyards. Regularly, different households with or without a family connection were sharing the same fenced courtyard and lived very close together.

#### Use of the home at night

The group members mentioned that commonly two to four people shared a room for the night in both rural and urban areas. Some people reported that their building or room had a partition of jutemade screen, dividing the room in two parts. Each room had one or two beds, which were shared with up to four people. When there were not enough places inside, family members slept on the verandah or shared a room with domestic animals.

#### Visitors to the home

Rural as well as urban participants mentioned that, during daytime, neighbours and those living in nearby villages visited one another daily. Visitors from outside the village or ward came less often. Male as well as female relatives of different ages were the most-frequently mentioned guests. When they lived far away, they often stayed for two to four nights. Sometimes, hawkers, businessmen, constructors, and day-labourers also stayed. A rural female adult reported:

Sometimes, a hawker from a different area comes to the village to sell different kinds of goods. When he needs to stay and asks for help, we give him a bed.

When guests stayed overnight, beds were shared with the visitors. This was done in both urban and rural areas. When there were many visitors and not enough beds were available, people slept on the floor. Bed-sheets, blankets, clothes, and towels were shared with visitors if necessary. Sharing of clothes was mentioned as a regular practice among household members in the rural areas. Boys shared clothes with their brother or father while girls shared with sisters, or their mother. In urban areas, sharing of clothes was not a common practice anymore. An urban adolescent girl reported:

Previously, two or three brothers used to share a set of clothes. Nowadays, people in this area are more modern and don't do that anymore. They wear their own personal clothes. Everyone wants to be different.

### Social contacts within the neighbourhood

People in rural as well as urban areas reported that they visited one another regularly in their own village or urban ward. Male adults and adolescents had the most opportunities to meet, in both rural and urban settings. Women reported to be more restricted to go out and meet other people, especially in urban areas. Everyone mentioned that they socialized most often with people of their own age and sex group.

Adult men mentioned that they spent most of their time outside the home for work. Rural men met with their co-workers in the fields under a tree or in a roofed meeting place near the fields when taking a break. After work, they often met at an open meeting place with a thatched or tin roof in the village, at the sport field, under a tree, with a common tubewell, or just on the streets of the village or ward. Adult and adolescent men from urban areas reported more opportunities to meet. They reported a wider variety of jobs in which they met with others while they also mentioned specific urban meeting places, like cinema halls and large meeting halls where events were organized.

Both urban and rural men met with their friends in the market areas since shopping was a task of the male household members in that region. A rural male adult reported:

At market places, we do business and talk to all kinds of people. If we meet our friends, we may go to a hotel to eat together or watch television.

Men also mentioned the mosque or temple as regular meeting places in their neighbourhood while social contacts at religious meetings and festivals were reported. Some religious meetings reported were on a small scale and lasted for a few hours but it was mentioned that, in all villages, large festivals lasting for three to five days took place once or twice a year, in which thousands of people gathered. The participants reported that many people sat closely together in small rooms on those occasions. In most Muslim meetings, men and women sat separately while Hindu men and women usually celebrated together.

They mentioned that fairs, weddings, and funerals were other gatherings in which many people of different ages and gender met. Most large gatherings took place outside or in a tent. The tents used for this kind of occasions were often (partly) open from the sides and were used only to protect people from the sun or rain.

Adult women in rural areas mentioned that they regularly met with neighbours and other women living nearby while doing household tasks in and around their home. They met and chatted under a tree, near a common tubewell or pond. They also met during their work in the fields. Many women worked closely together in groups. A rural female adult reported:

We seven women work together at the same place, we work in the jute field, cut and bind tobacco or cut paddy. During our work, we can chat with one another. We work from dawn to dusk and stay together all day long. We do our work together and have our snack, and lunch together. Women work together, while men work separately.

Many participants in the rural locations reported not to have their own television. They mentioned that men, women, and children watch television together in someone's home and that those places were often very crowded, especially when a popular programme or movie was shown. Women in both urban and rural areas met with others at special occasions, like weddings, religious meetings (Muslim and Hindu), and fairs. In all discussion groups, women mentioned that they took part in several of those occasions. A rural female adult reported:

Once a month, a pious person comes to talk on religious issues in our Hindu temple. When we have the opportunity, we attend the programme. We also have different types of fairs. The fair of ‘Mangol Protima’ has just started this Tuesday. Many people attend this fair. It is so crowded that it is difficult to move from one place to another.

A Muslim urban female adult reported:

Besides Jumma prayers, which take place every week on Fridays and in which 40/50 women participate, we regularly attend special ceremonies. Often, we have to sit very closely together as these ceremonies are very crowded.

A Hindu urban female adult reported:

Yearly, we have two fairs in our temple in which thousands of people attend. Everyone comes, both Hindu and Muslim. There are also cultural programme in which local artistes perform. People from other districts also come to our fairs.

Many women also took part in religious or micro-credit associations. They reported to have meetings on a regular basis for this purpose at a house or at an NGO office in the neighbourhood. In most of these meetings, only women participate.

Contrary to urban men, urban women reported to have fewer opportunities to meet than rural women. Many of them mentioned restrictions to go outside alone and limited contacts. None of the urban adult women was working outside the home while, in the adolescent group, only one girl mentioned that she was working in a beauty parlour. Women spent most of their time inside or around their home. Contrary to rural women, they reported not to watch television in common places but had their own television and watch at home. Meeting places mentioned by urban women were: the school while bringing their children, the beauty parlour, or the Hindu temple during their weekly visit for praying.

Adolescent boys and girls mentioned that they spent most of their time in school. They mentioned that most of the schools were mixed in the area, with boys and girls attending the same class. Children sat closely together in benches or on mats on the floor during the lessons. They went to school for 6 days a week. A rural adolescent girl reported:

Most of our time, on average 6 hours, we spend in school. The number of students differs from class to class. It can range from 40 to 100 students, both boys and girls. In all classrooms, 4 students share one bench.

Adolescents reported to spend most of their time, after school, with friends of the same age and sex group. Boys mentioned that they played football or cricket, swam in the pond, chatted at central meeting places and went to clubs, hotels or tea-stalls to play the board game ‘carrum’ or watch television. Some of them mentioned they went to cinema halls to watch movies. Both boys and girls mentioned they visited religious festivals, fairs, and cultural programmes within their village and met with their friends on those occasions.

Adolescent girls reported more restrictions to go out than boys of the same age and spent more time in and around their home. Girls in the urban area reported even more restrictions than girls living in the rural villages. However some of them mentioned that they visited female friends in other families living nearby to chat or to celebrate birthdays. Some girls mentioned that they were not allowed to visit friends or go outside alone. An urban adolescent girl reported:

Sometimes, I go to a friend's home and meet there with 3-4 friends for 1½-2 hours. However, a family member always accompanies me.

Another urban girl reported:

I am not allowed to go outside to visit my friends; therefore, I always stay at home after school.

### Social contacts outside the neighbourhood

The rural women in the discussion groups mentioned visits to relatives in nearby villages and the nearby town but none of them ever left the district. Some of the urban women mentioned that they visited relatives in other towns in Bangladesh but this happened occasionally. None of them ever left the country.

Rural male adolescents mentioned that they regularly visited nearby towns for shopping or going to school in the city. They also went to other districts to visit relatives or to work. The rural adult men who participated in the focus group discussions mentioned that they went to other districts to work as rickshaw-puller or day-labourer in agricultural or factory work in periods of shortage of money. They shared a room with many other people under poor hygienic conditions. Only two rural men went outside the country once. They both went to India.

Urban men mentioned that they travelled to other areas in Bangladesh on a regular basis, to work, to do business, or to attend religious meetings and sport events. Two urban adult men reported that they travelled to India every 6 months to visit their relatives. Urban men mentioned that, in case they visited other areas for a longer period of time, they stayed with relatives or in a shared room in a guesthouse or hotel. Many of them reported that they shared their room with many other people in the past. An urban male adult reported:

When we go to Dhaka with our sports team, we stay with 20-25 people in a room on the floor. People come from other upazillas too. We stay very close, as in a wedding programme.

### Relevance of contact patterns for the transmission of airborne infectious diseases

Social contact patterns as reported during the group discussions were ordered by distance from home, the core unit for social contacts (in the home, within the neighbourhood, and outside the neighbourhood) and by perceived relevance for the transmission of airborne diseases (Table 2).

Inside the home, social contacts highly relevant for the transmission of airborne infectious diseases were reported in all discussion groups. Outside the home, contact patterns were assortative with regard to age and sex. Women and girls reported relevant social contacts limited to the neighbourhood while men reported social contacts relevant for the transmission of airborne diseases outside their neighbourhood as well (Table 3).

**Table 2.** Social contact patterns in rural Bangladesh with relevance to the transmission of airborne infectious diseases

Relevance for disease transmission (Distance from the home)	High relevance	Medium relevance	Low relevance
Inside the home	- Sharing of bedroom and/or bed with someone: physical contact - Sharing clothes and towels - Spending time inside the home with household members - Meeting inside the home with visitors for more than half-a-day; visitors who stay overnight - Daily meetings with the same group of people: neighbours or friends living nearby	- Meeting with visitors inside the home occasionally or for less than half-a-day - Contact with household members in the courtyard	- Meeting with visitors in the courtyard
Within the neighbourhood (village or urban ward)	- Contact inside a small room for more than half-a-day or regularly - Meeting inside a crowded large room, meeting hall, or closed tent for long period: religious festivals, wedding, other special occasions - Daily meeting in a small room or crowded large room: workplace, school	- Incidental meeting inside a small room or building for less than half-a-day: watching television, meeting friends in a restaurant, shop, or beauty parlour - Incidental meeting inside a large room or meeting hall with many people for less than half-a-day: temple, mosque, community meeting hall - Regular (at least weekly) meeting in a relatively large room or meeting place: meeting of associations or other community groups	- Daily meetings in outdoor places: outdoor meeting places in the village/neighbourhood, meeting for work in the field - Meeting in places visited for a short time: shop, market, school area to collect children - Incidental outdoor occasions with many people: outdoor festivals and fairs
Outside the neighbourhood	- Staying overnight in another area and share a room and/or bed with others - Staying overnight in a room with more than 10 other people - Sharing clothes or towels - Meeting people inside a small room or house for more than half a day: relatives or friends on special occasions - Meeting inside a large room, closed tent or meeting hall with many people for more than half-a-day: work, religious festivals, wedding	- Meeting with other people inside a small room or house for less than half-a-day: business meetings, short visits of relatives and friends - Meeting with people inside a relatively large room or meeting hall	- Meeting people in places visited for a short time: shop, market, etc. - Outdoor occasions with many people, like outdoor festivals and fairs - Meeting people during outdoor work

**Table 3.** Contact patterns with high relevance for transmission of airborne infectious diseases: pattern reported by different subgroups

Distance from the home	Contact pattern	Female				Male			
		Rural adult	Rural adolescent	Urban adult	Urban adolescent	Rural adult	Rural adolescent	Urban adult	Urban adolescent
Inside the home	- Sharing bedroom and/or bed: physical contact	+	+	+	+	+	+	+	+
	- Sharing clothes and towels	+	+	-	o	+	+	o	o
	- Daily contact with household members	+	+	+	+	+	+	+	+
	- Daily contact with neighbours and friends living nearby	+	+	+	+	+	+	+	+
	- Visitors who come to stay overnight	+	+	+	+	+	+	+	+
Within the neighbourhood (village or urban ward)	- Regular (at least weekly) meetings with others inside small rooms or houses	+	+	+	+	+	+	+	+
	- Daily meeting inside a room or building (workplace, school)	-	+	-	+	-	+	+	+
	- Regular meetings inside crowded large rooms, meeting halls, mosque or temple or closed tents	o	-	o	o	+	+	+	+
	- Incidental meetings in crowded large rooms, meeting halls, mosque or temple or closed tents for long period	+	+	+	+	-	+	+	+
Outside the neighbourhood	- Travelling to nearby villages and towns	o	o	o	-	-	+	+	+
	- Travelling to other districts	-	-	o	-	+	+	+	+
	- Travelling outside the country	-	-	-	-	o	-	o	-
	- Staying overnight in other areas	-	-	o	-	+	o	+	+
	- Spending the night with more than 10 people in one room	-	-	-	-	+	-	+	o

+=Mentioned by more than 25% of the group or reported as common behaviour; o=Mentioned by less than 25% of the group; -=Reported as not present in this group

## DISCUSSION

Social contact patterns are shaped by the social environment and influenced by cultural habits and economic circumstances. An exploration by focus group discussions in northwest Bangladesh revealed that, in this region, intergenerational contact and contact among people of different sex groups are common in the home and nearby neighbourhood. Outside the home, there are marked differences by gender. Women and girls have social contacts relevant for the spread of infectious diseases, like leprosy and tuberculosis that are transmitted from person to person around the home only. Men have highly relevant contacts beyond the home. This implies that, in theory, these diseases can be spread easily across age and sex groups in and around the home while adult men can be involved in the spread from outside this relatively confined area.

Strength of this study was that we ordered social contact patterns as explored by means of focus group discussions by perceived relevance for the spread of airborne infectious diseases. We did a broad exploration on how people interact since knowledge on contact patterns was limited for northwest Bangladesh, especially in relation to the spread of infectious diseases.

By selecting the participants, we assured that the two main religious groups in the region (Hindu and Muslim) were represented in each age/sex group to cover practices of both the groups. This resulted, however, in a relatively high percentage (55%) of Hindus while only 10% of the general population of the area is Hindu.

A limitation of the study design is that people tend to mention only socially-acceptable contact patterns in a group discussion setting. Exceptional ideas or habits are not always shared. We tried to overcome this problem by choosing a comfortable setting with enough privacy and by using facilitators of the same sex as the participants, who were familiar with the area and local customs.

Another limitation of the use of a qualitative study design is that it is not suitable to establish a causal relationship between social determinants and airborne infectious diseases. Therefore, further studies are needed to quantify the results and establish causal relationships.

Despite these limitations, participants of the focus group discussions reported a consistent pattern of social contacts, in line with the expert opinion of the local health staff and with the limited published information on social contact patterns in Bangladesh. As in other published material regarding social networks in Bangladesh, households were identified as the place where the most intensive social contacts take place across different age and sex groups while women reported a lower mobility than men due to cultural customs (22,23).

There are not many published studies actually addressing social contact patterns in relation to transmission of airborne infectious diseases. Although studies were carried out in Viet Nam and South Africa, most were conducted in high-income countries with different cultural practices than in our study area. However, the contact profiles and implications for transmission of infectious diseases have similarities with the results of our study. In all studies, households were identified as the most important connective place for people of different age and sex groups (26-28). Two studies concluded that households play an important bridging role in the transmission of airborne diseases among population subgroups (26,28). In a study in South Africa, the same role was assigned to public transport where intergenerational mixing also takes place (28). As in our study, several other studies indicated that social contacts outside the home are highly associated with age and sex (14,27,29). Because meeting places depend highly on the social environment, there are also major differences among the studies. Contacts during leisure activities, for example, were important in Europe but not in Viet Nam and South Africa while public transport was only important in South Africa.

Our study in Bangladesh is unique in showing a very marked difference in social contact patterns by gender. This brings about different risks of getting infected with airborne infectious diseases for male and female. These might be accountable for the differences in sex ratio for both tuberculosis and leprosy since male cases are reported more frequently than female in Bangladesh and other Asian countries (30,31). Contrary, no differences by gender are observed in Africa and South America and regions with other sociocultural norms and practices. Underreporting in females was mentioned as possible explanation in the Asian region (32), though not the same infection risks due to different social contact patterns might also play a role.

Of particular interest are the social contacts adult men reported outside their neighbourhood. In periods of the shortage of money, poor men reported that they went to other areas to work as labourer or rickshaw-puller and stayed there for several weeks or months, with many people together in small rooms under deplorable hygienic conditions. There is a high risk of transmission of airborne infectious diseases under these circumstances.

Another interesting fact was that people reported to live closely with domestic animals in this area of Bangladesh. Several people reported to sleep in the same room as their animals. Although this practice does not increase the risk of person-to-person spread of diseases among humans, it entails an increased risk for the spread of zoonoses, of which outbreaks are regularly reported from Bangladesh, for example, bovine tuberculosis, anthrax, avian influenza, or Nipah virus (33-35).

Though not conclusively, the framework of relevant social contact patterns as presented can be used as a start for further quantitative exploration on the association between social contact patterns and airborne infectious diseases in this region. The concepts revealed should be explored further but can be important for the control of infectious diseases in Bangladesh and other countries in South Asia. Control programmes in this region could benefit from considering differences in social contact patterns by gender, for risk assessments and planning of preventive interventions.

## ACKNOWLEDGEMENTS

The authors want to thank the staff of the Rural Health Program in Nilphamari for their dedication and hard work in organizing and conducting the focus group discussions, especially for transcribing the data. Special thanks are also due to the staff of icddr,b, who translated the transcripts into English.
